# Vallecular Cyst: Reminder of a Rare Cause of Stridor and Failure to Thrive in Infants

**DOI:** 10.7759/cureus.19692

**Published:** 2021-11-18

**Authors:** Amal Alnaimi, Ahmed Abushahin

**Affiliations:** 1 Pediatric Pulmonology, Sidra Medicine, Doha, QAT

**Keywords:** respiratory distress, laryngoscopy, failure to thrive, stridor, vallecular cyst

## Abstract

The vallecular cyst is a rare cause of stridor, respiratory distress, and failure to thrive in infants. Large vallecular cysts may present with serious complications such as life-threatening airway obstruction. This report is of an infant who presented with stridor and failure to thrive. The patient’s condition was diagnosed as the presence of a vallecular cyst using flexible laryngoscopy. The vallecular cyst was successfully managed using endoscopic marsupialization. After the procedure, the patient was asymptomatic and dramatically gained weight within a few months. This case report serves as a reminder for clinicians to consider vallecular cysts as a differential diagnosis of stridor and failure to thrive in infants. It also emphasizes that early diagnosis and management lead to favorable clinical outcomes.

## Introduction

Vallecular cysts are rare, representing 10.5-20.1% of all congenital laryngeal cysts [1-2]. The overall incidence rate of these cysts is approximately 3.49-5.3 cases per 100,000 newborns [2-3]. Clinical features include stridor, respiratory distress, poor feeding, and failure to thrive [4-5]. Larger lesions may lead to life-threatening airway obstructions, which is of greater concern [6].

Herein, we present a case of vallecular cyst in an infant who presented with stridor and failure to thrive; the patient’s condition markedly improved, and no complications occurred owing to the appropriate diagnostic procedure and treatment approach. We emphasize the importance of considering a vallecular cyst as a differential diagnosis for stridor and failure to thrive in infants and highlight that prompt diagnosis and proper management of vallecular cysts lead to favorable clinical outcomes.

## Case presentation

A three-month-old male infant presented with stridor and failure to thrive. He was delivered vaginally at full-term (birth weight 3.5 kg) and had an uncomplicated neonatal course. His parents reported that he had noisy and difficult breathing a few days after birth, which worsened over time and was associated with episodes of cyanosis and poor bottle feeding. A general pediatrician saw him at one month of age for stridor and poor weight gain, assumed a diagnosis of laryngomalacia, and advised the parents to increase the frequency of his feed. After that, his parents sought medical advice several times, including emergency department visits for significant respiratory distress, increasing stridor, and failure to thrive. He was then referred to our neurology clinic to assess hypotonia. No workup was performed, and he was not hospitalized before the referral. Physical examination revealed inspiratory stridor, suprasternal and subcostal retractions, tachypnea, and bilaterally reduced air entry. His oxygen saturation, which was 93% in room air, and improved slightly after oxygen supplementation. His weight at presentation was 4.2 kg, falling below the 3rd percentile of the WHO growth chart. No dysmorphic features were present.

**Figure 1:**
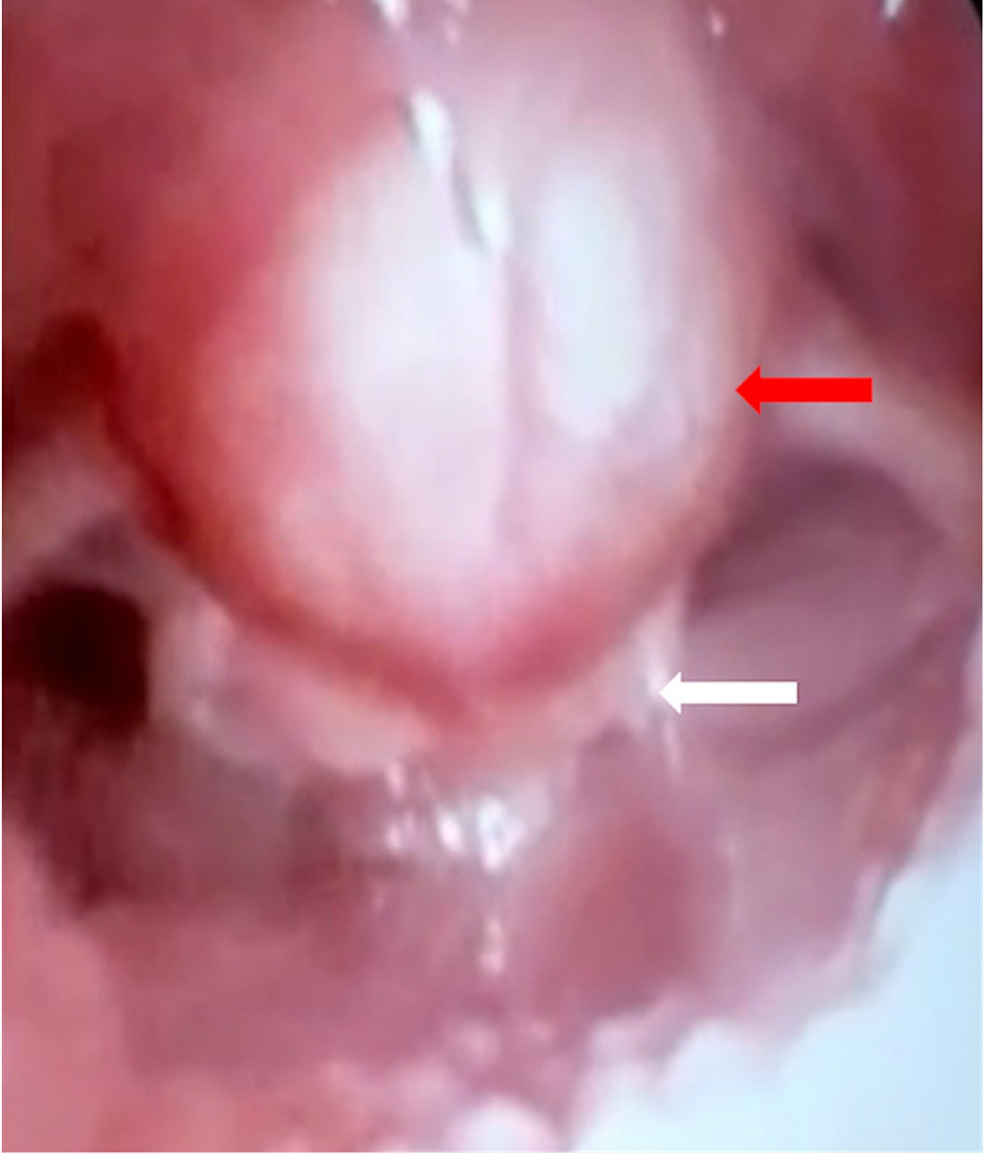
Flexible endoscopic view of the patient’s vallecular cyst (red arrow) The cyst is displacing the epiglottis (white arrow) and obstructing the airway.

Chest X-ray and regular laboratory test findings were normal, including serum electrolytes, complete blood counts, renal and liver functions, thyroid hormone levels, and blood gas analysis. The patient was taken to the operating room for an airway assessment based on the above findings. The patient underwent flexible laryngoscopy, which revealed a cystic mass measuring approximately 2 x 3 cm in size, arising from the lingual surface of the epiglottis and significantly occluding the laryngeal inlet (Figure 1).

Direct laryngoscopy and bronchoscopy under general anesthesia were promptly performed to evaluate the extent of the cystic lesion. The vocal cords, subglottic area, and trachea were observed to be normal. A thyroglossal cyst and retention cyst were initially considered as the differential diagnoses. However, based on clinical, endoscopic, and later histological findings, a vallecular cyst was established as the final diagnosis.

Microlaryngoscopic marsupialization was performed by the ENT surgeon to remove the cyst, which drained serous fluid. A biopsy of the cyst wall revealed the presence of connective tissue covered with non-keratinizing squamous epithelium with vascular congestion and chronic inflammation, suggestive of a vallecular cyst.

Postoperatively, the patient was feeding well and showed a complete resolution of his symptoms. He remained asymptomatic at the follow-up visits at four and seven months. He quickly gained weight within a few months, reaching 5.5 kg at four months and 8.4 kg at seven months of age, indicating that the child’s weight had increased from below the 3rd percentile to the 50th percentile.

## Discussion

The vallecular cyst is a rare cystic lesion but a well-recognized cause of stridor, respiratory distress, and failure to thrive in infants due to obstruction at the laryngeal inlet [1-3]. Vallecular cysts result from mucus retention within mucous glands due to ductal obstruction or an embryological defect [7]. Histologically, they contain respiratory epithelium and mucous glands within an external squamous epithelial layer [8]. These cysts develop underneath the mucosa of the vallecula, typically on the lingual surface of the epiglottis, as seen in our case, or at the base of the tongue [2,4].

Vallecular cysts contain serous fluid and are usually less than 1 cm; however, further expansion may occur with mucus production [1,4]. Infants typically present with varying degrees of breathing and feeding difficulties within the first few weeks of life with a median age at diagnosis of three to 40 days [2]; the severity of symptoms depends on the cyst’s size and extension and the presence of concomitant airway anomalies like laryngomalacia [4,6]. For slowly growing cysts, clinical features may only occur at a later age [9]. Warning clinical features for the VC include progressive stridor, respiratory distress, apneic episodes, cyanosis, poor feeding, and failure to thrive [4-5]. Hence, laryngeal lesions, including VC, can be suspected based on a complete history and a thorough physical examination, particularly when an infant presents with congenital and progressive stridor. Our patient presented with progressive stridor, poor feeding, and failure to thrive, with his weight dropping below the 3rd percentile.

The most serious complication is respiratory failure or death due to an upper airway obstruction, which is more likely to occur when the cyst is large and close to the laryngeal inlet [5-6].

Anomalies that may complicate the clinical course of vallecular cysts include laryngomalacia and gastroesophageal reflux disease (GERD), which have been reported in 17% and 67% of cases, respectively [8,10]. However, neither of these anomalies were observed in our case.

Diagnosis of vallecular cysts demands a high level of clinical suspicion. When a vallecular cyst is suspected in infancy, a lateral radiograph of the neck may show an alteration of the airway contour. However, Suzuki et al., in their series of VC cases, found that lateral neck X-ray did not contribute to the diagnosis in 15 of the 39 VC cases [4]. These findings suggest that lateral neck X-rays are inadequate and have a limited contribution to the diagnosis of congenital stridor.

The definitive diagnosis is made by directly visualizing the vallecular cyst using fiberoptic nasal endoscopy, as was performed in our case, or with rigid laryngoscopy/bronchoscopy [10-11]. Computed tomography and magnetic resonance imaging are helpful in the preoperational setting; they can delineate the cyst’s structure, vascularity, and its extension and can identify other lesions such as hemangioma and thyroglossal cyst [4,11]. Other cystic lesions that cause stridor such as lingual thyroid, thyroglossal duct cyst, lymphatic malformation, or teratoma should be considered in the differential diagnosis of a vallecular cyst [8,11]. 

Treatment options include complete endoscopic excision, marsupialization, or aspiration. Marsupialization is the preferred treatment modality and may be performed using micro-laryngeal instruments, a CO_2_ laser, cauterization, or coblation [2,11]. Coblation-assisted cyst removal is a novel and effective minimally invasive procedure [7]. The recurrence rate following marsupialization is neglectable [8]; Suzuki et al. reported a recurrence rate in one of 39 patients after marsupialization, and none of 14 patients underwent laser marsupialization [4]. Cyst aspiration is not recommended because it increases the risk of cyst recurrence; however, it can be considered in emergencies, such as severe airway obstruction, before attempting complete removal of the cyst [11]. Our patient underwent marsupialization via micro-laryngeal instruments and showed a significant improvement in symptoms with no postoperative complications.

## Conclusions

Small vallecular cysts of less than 1 cm may cause mild symptoms while larger ones may cause life-threatening airway obstruction. Physicians should consider a vallecular cyst as a differential diagnosis of stridor and failure to thrive in infants. Early identification and removal of a vallecular cyst result in its definitive cure and may prevent its associated serious consequences.
